# An insect virus differentially alters gene expression among life stages of an insect vector and enhances bacterial phytopathogen transmission

**DOI:** 10.1128/jvi.01630-24

**Published:** 2024-12-23

**Authors:** Chun-Yi Lin, Jacobo Robledo Buritica, Poulami Sarkar, Ola Jassar, Sâmara Vieira Rocha, Ozgur Batuman, Lukasz L. Stelinski, Amit Levy

**Affiliations:** 1 Citrus Research and Education Center, University of Florida 57513 , Lake Alfred, Florida, USA; 2 Department of Plant Pathology, University of Florida 316814 , Gainesville, Florida, USA; 3 Agricultural Research Organization, Volcani Center 530612 , Rishon LeTsiyon, Israel; 4 Department of Genetics and Evolution, Federal University of São Carlos 236041 , São Carlos, Brazil; 5 Department of Plant Pathology, Southwest Florida Research and Education Center, University of Florida 53710 , Immokalee, Florida, USA; 6 Entomology and Nematology Department, University of Florida 166763 , Gainesville, Florida, USA; Iowa State University, Ames, Iowa, USA

**Keywords:** *Diaphorina citri* flavi-like virus, *Candidatus *Liberibacter asiaticus, acquisition efficiency, transmission

## Abstract

*Diaphorina citri* transmits *Candidatus* Liberibacter asiaticus (CLas) between citrus plants which causes the expression of huanglongbing disease in citrus. *D. citri* flavi-like virus (DcFLV) co-occurs intracellularly with CLas in *D. citri* populations in the field. However, the impact(s) of DcFLV presence on the insect vector and its interaction with the CLas phytopathogen remain unclear. We compared CLas acquisition and transmission efficiencies as well as transcriptomic expression between viruliferous and non-viruliferous psyllids at multiple life stages. Viruliferous nymphs acquired higher titers of CLas than non-viruliferous nymphs, whereas viruliferous adults acquired less CLas than those without virus. The presence of DcFLV increased the transmission of CLas by both nymphs and adults. Furthermore, RNA-seq and functional gene expression analyses revealed that endoplasmic reticulum stress-, autophagy-, and defense-related genes were significantly upregulated in viruliferous adult psyllids, whereas most of these genes were downregulated in viruliferous nymphs. Our work demonstrates that DcFLV differentially modulates various cellular and physiological functions in *D. citri* in a life stage-dependent manner and promotes the acquisition of CLas at the nymphal stage and transmission of the pathogen at the adult stage of the vector. Collectively, our results suggest that *D. citri* vectors with DcFLV exhibit greater pathogen transmission efficiency than those without virus.

**IMPORTANCE:**

Huanglongbing (HLB), caused by fastidious bacteria from three *Candidatus* Liberibacter species, is the most damaging disease impacting the citrus industry worldwide. Spread by the Asian citrus psyllid (*Diaphorina citri*) in Asia and the Americas, HLB causes substantial financial losses, and has reduced citrus production in Florida by more than 90%. Although there are ongoing efforts to limit spread of the disease, effective HLB management remains elusive. Suppressing vector populations and decreasing CLas transmission are the two strategies that need to be urgently improved. Recently, a *D. citri* flavi-like virus (DcFLV) was characterized within its *D. citri* host, and it co-occurs intracellularly with CLas in psyllid populations. Here, we show that viruliferous nymphs exhibit higher CLas acquisition than non-viruliferous nymphs. Furthermore, both viruliferous adults and nymphs exhibit increased CLas transmission efficiency. We suggest the possibility of manipulating DcFLV in *D. citri* populations to reduce CLas transmission for HLB disease management.

## INTRODUCTION

Asian citrus psyllid, *Diaphorina citri* Kuwayama (Hemiptera: Liviidae), is the insect vector of the fastidious, phloem-limited bacterium, *Candidatus* Liberibacter asiaticus (CLas), which is one of the causal agents of citrus huanglongbing (HLB) worldwide. HLB has spread quickly and impacts all commercial citrus cultivars, causing severe reductions in fruit quality, yield, and lifespan of infected trees (1). In the United States, this devastating pathogen has spread throughout all major citrus-growing regions in several states, including Florida, California, and Texas (2). In the Florida citrus industry alone, citrus production has decreased by 90% since the initial identification of HLB disease in the state (3, 4).

CLas is circulative and propagative within the *D. citri* vector, entering the hemolymph through the gut barrier, and must reach the salivary glands and replicate to high titers before it can be transmitted during subsequent psyllid feeding at a latent period of 1–25 days (5, 6). The successful spread of CLas by *D. citri* depends on the efficiencies of pathogen acquisition and inoculation. The *D. citri* vector is a hemimetabolous insect with three development stages: egg, five nymphal instars, and adult (7). Longer and more frequent feeding times, and higher rates of CLas acquisition (60–100%) have been observed to occur during the nymphal stages than during the adult stage (8, 9). Nymphs also harbor higher titers of CLas than adults, and CLas transmission occurs more efficiently if adults acquire the bacterium as nymphs rather than during the adult stage (10–12). However, the impact of insect-specific viruses (ISVs) on the HLB pathosystem is still poorly understood. The *D. citri* flavi-like virus (DcFLV) is a positive-sense, single-stranded RNA virus with a genome of 27 kb in length, containing a single open reading frame. It represents the largest genome among known flavi-like viruses and shares genome organization and encoded proteins with viruses from the family Flaviviridae (13). DcFLV is one of five *D*. *citri*-associated viruses with an occurrence rate ranging between 13.71% and 20.83% in *D. citri* populations among six major citrus production areas in Florida (14). Lin et al. (15) and Rashidi et al. (16) demonstrated that: (i) DcFLV and CLas co-occur in psyllid populations; (ii) CLas titers are higher in nymphal psyllids within populations that are co-infected with DcFLV than in those without virus; and (iii) both microbes are localized within the midgut and also occur in the salivary gland cells. These results suggest an interaction between the two microbes and highlight knowledge gaps about how DcFLV may affect the epidemiology of HLB.

Manipulation of insect-associated microbes or endosymbionts may be a viable approach to manage the spread of vector-borne pathogens. Insect-borne pathogens can indirectly affect their own acquisition by vectors, modulate plant defenses, and/or alter host-plant chemistry (17, 18). Martini et al. (19) demonstrated that CLas may manipulate vector movement and mate selection by increasing the probability of its dispersal and the attractiveness of infected female insects. During HLB disease development, an association was observed between citrus odor preference by psyllids and CLas-mediated plant volatile emissions (20). Mann et al. (21) also showed that CLas induces changes in citrus plant volatile metabolites, and that the specific resulting odorants can mediate psyllid preference, indicating that this host-mediated manipulation of vector preference may facilitate pathogen spread. In addition, parasite or endosymbiont infection can alter several insect host traits by affecting development, morphology, and behavior (22–25). For instance, occurrence of the endosymbiotic bacterium, *Wolbachia*, within insects is known to promote or reduce various behaviors and physiological processes, such as mating or feeding frequency (26–28). In *D. citri* co*-*infected with *Wolbachia* and CLas, the expression of phage lytic cycle genes is repressed in the CLas bacterium as compared with that of CLas occurring in psyllids without *Wolbachia* (29). In contrast, reducing titers of *Wolbachia* in *D. citri* increases CLas acquisition but decreases fitness of the insect (30). To date, the influence of *D. citri*-associated viruses on psyllid behavior and physiology is unknown and the interactions among the virus, CLas phytopathogen, and insect vector have not been described.

In the present work, we investigated how the presence of DcFLV affects subsequent acquisition and transmission efficiencies of the CLas phytopathogen by *D. citri*. We demonstrate that virus infection differentially alters vectorial capacity of the psyllid, and this effect depends on the specific life stage at which psyllids become infected with the virus. We also compared levels of functional gene expression between viruliferous and non-viruliferous nymphs and adults in an effort to elucidate potential molecular mechanisms explaining the effect of virus infection on vector competence.

## RESULTS

### Presence of DcFLV increased acquisition of CLas by *D. citri* nymphs but not by adults

**Fig 1:**
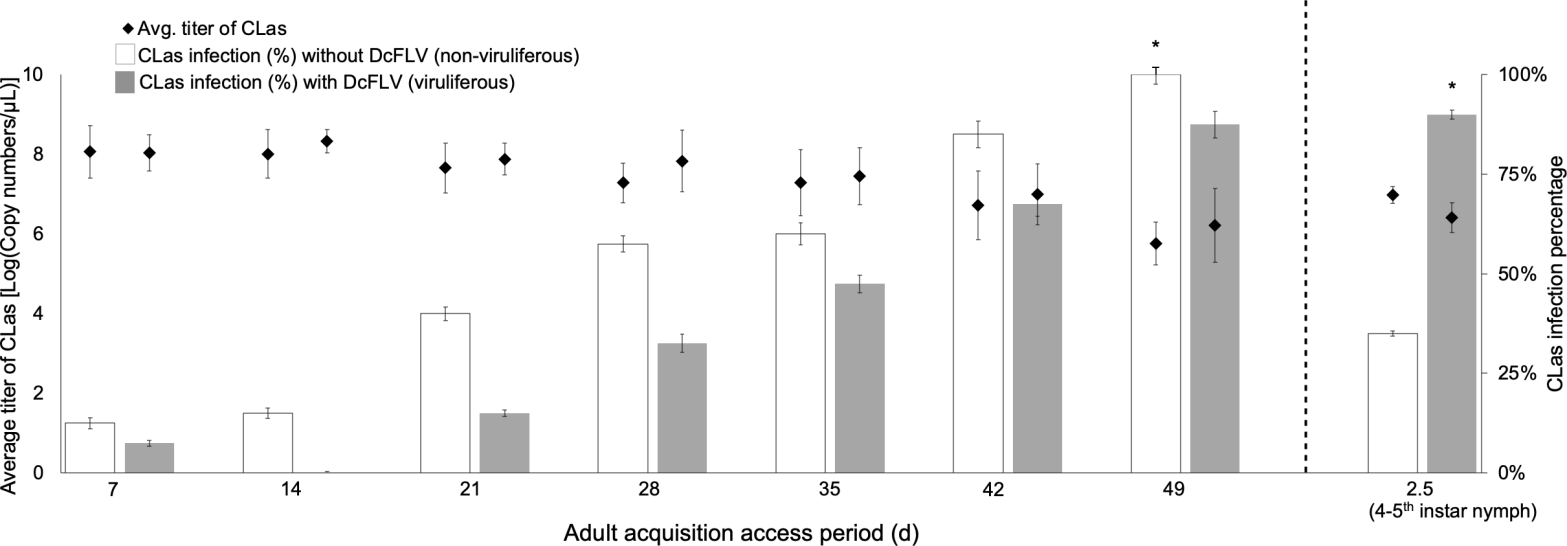
Percentage of CLas-positive *Diaphorina citri* adults after acquisition access periods (AAPs) of 1–49 days, and fourth- and fifth-instar nymphs after AAPs of 2.5 days, with or without DcFLV presence on CLas-infected citrus plants. CLas presence and titer were quantified by qPCR following each AAP. All data were analyzed by Student’s *t* test, and the asterisks represent statistically significant differences (*P* < 0.05).

Following acquisition access periods (AAPs) of 7, 14, 21, 28, 35, 42, or 49 days for adults, the acquisition of CLas by viruliferous adults was generally lower across all AAPs compared to that of non-viruliferous adult counterparts. A significantly lower percentage of viruliferous *D. citri* adults acquired CLas at significantly lower titers (87.5%; avg. Ct: 30.0; avg. copy number/μL: 8.5E4) than non-viruliferous adults (100%; avg. Ct: 28.3; avg. copy number/μL: 1.6E5) at 49 days, indicating that CLas acquisition efficiency was lower among viruliferous than virus-free adults (Student’s *t* test: *P* < 0.001) (Fig. 1). In contrast, following 2- or 3-day AAPs for nymphs, viruliferous nymphs exhibited significantly higher acquisition efficiency (90%; avg. Ct: 30.8; avg. copy number/μL: 2.3E4) compared with non-viruliferous nymphs (35%; avg. Ct: 33.1; avg. copy number/μL: 5.6E3) (Student’s *t* test: *P* < 0.01) (Fig. 1). Various AAPs were employed to determine if a consistent trend was present across different durations of pathogen acquisition, thereby reinforcing our findings, which demonstrated a consistent significant difference between viruliferous and non-viruliferous adults over time. The results indicated that presence of DcFLV decreased CLas acquisition by adult psyllids following AAPs ranging between 7 and 49 days. However, fourth- to fifth-instar nymphs acquired more CLas in the presence of DcFLV than when virus was absent.

### Presence of DcFLV increased CLas transmission efficiency by both adult and nymphal *D. citri*

**Fig 2:**
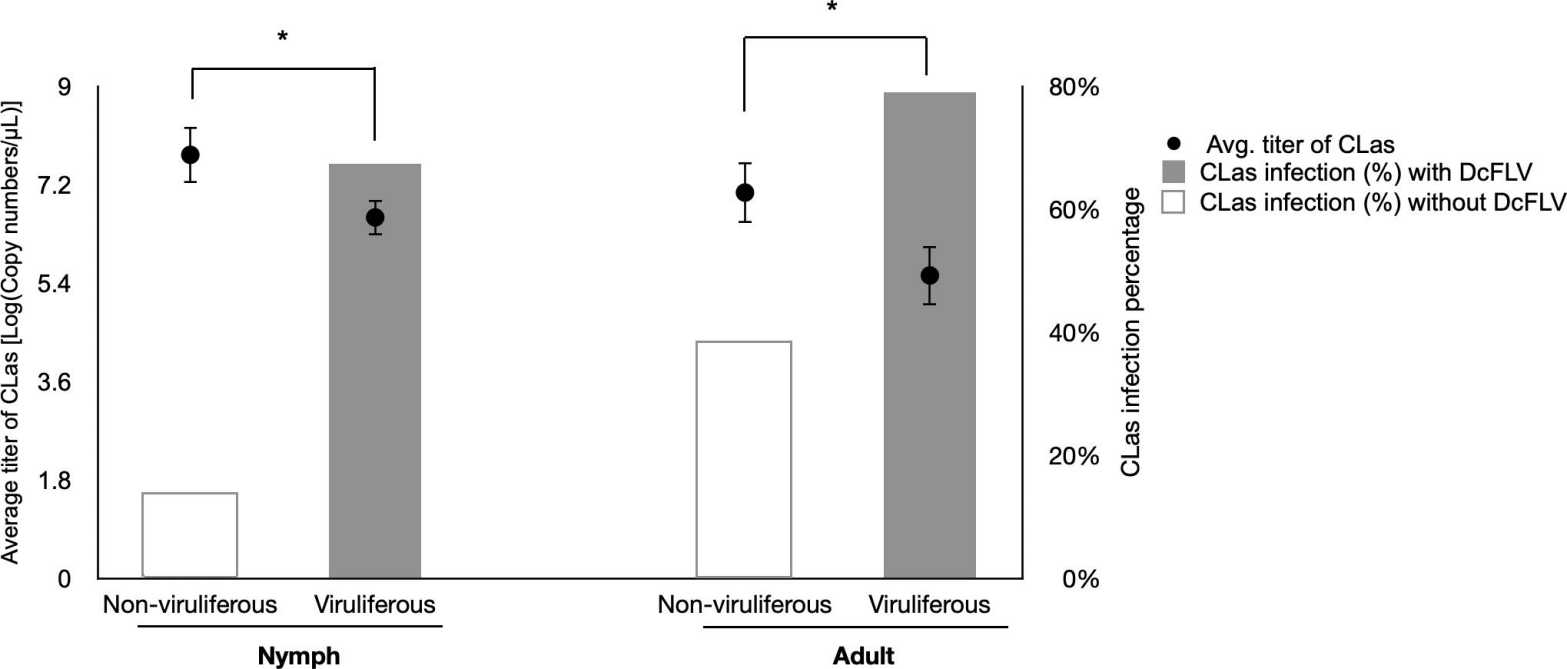
Bar and dot graphs representing the percentage of *Diaphorina citri* nymphs or adults infected with CLas and the average CLas titer in psyllids, respectively, after inoculation access periods by both viruliferous and non-viruliferous psyllids. Values represented by the dot graph are mean ± SD. The asterisks represent statistically significant differences obtained by Student’s *t* tests (*P* < 0.05).

In the transmission experiment, a total of 84 *C*. *macrophylla* seedlings were exposed to non-viruliferous (41 seedlings) or viruliferous (43 seedlings) nymphs. A total of 57 *C*. *macrophylla* seedlings were inoculated by non-viruliferous (26 seedlings) or viruliferous (31 seedlings) adults. The percentages of CLas-infected plants at 3, 6, and 9 months after inoculation are presented in Table 1. For nymph inoculations, viruliferous nymphs exhibited significantly higher CLas transmission efficiencies (14–23% of plants infected) than non-viruliferous counterparts during all testing periods. Similar results were observed with viruliferous adults causing higher infection percentages (12.5–41.7% of plants infected) than non-viruliferous counterparts at 6- and 9-month post-psyllid inoculation. CLas titers and infection percentages in nymphs and adult insects were also determined after the inoculation access periods (IAPs) (Fig. 2). Both viruliferous nymphs and adults were characterized by significantly higher CLas titers (Ct values: 31.2; avg. copy number/μL: 4.3E5 and 27.2; avg. copy number/μL: 3.6E6) than that found in non-viruliferous counterparts (Ct value: 36; avg. copy number/μL: 2.2E5 and Ct value: 33.5; avg. copy number/μL: 2.3E6) (Student’s *t* test: *P* < 0.001). Also, a higher percentage of both viruliferous nymphs and adults were infected with CLas (67.4% and 79%, respectively) than non-viruliferous counterparts (13.9% and 38.2%, respectively).

**TABLE 1:** CLas transmission by nymph or adult *Diaphorina citri* with or without the presence of DcFLV

	3 m.a.i.^*a*^	*χ²*	*P* ^*b*^	6 m.a.i.	*χ²*	*P*	9 m.a.i.	*χ²*	*P*
Non-viruliferous nymph	2.4% (1/41)	8.9377	0.0027**	5.1% (2/39)	4.5849	0.0322*	6.7% (1/15)	10.5059	0.00118**
Viruliferous nymph	14.0% (6/43)			14.0% (6/43)			23.0% (8/35)		
Non-viruliferous adult	4% (1/25)	0.0218	0.8823	4.3% (1/23)	4.3694	0.03658*	17.6% (3/17)	13.9224	0.00019***
Viruliferous adult	3.6% (1/28)			12.5% (3/24)			41.7% (10/24)		

^*a*^ m.a.i., month after inoculation.

^*b*^ *Significant at <0.05 level; **significant at 0.01 level; ***significant at 0.001 level. Differences determined by *χ²* analysis.

### Presence of DcFLV upregulated more differentially expressed genes and induced more differential enrichment pathways in *D. citri* adults than in nymphs

**Fig 3:**
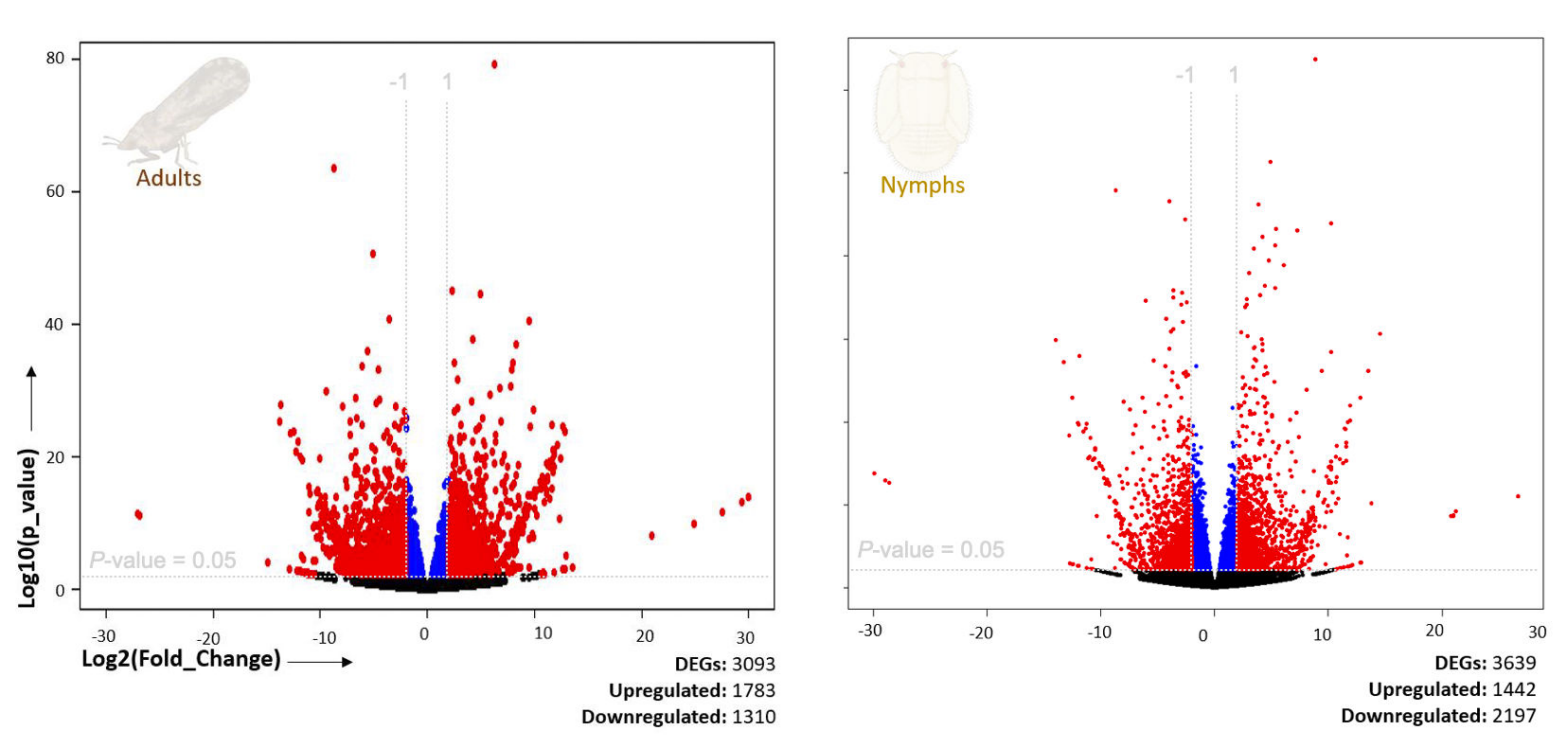
Volcano plots of differentially expressed genes (DEGs) in *Diaphorina citri* nymphs or adults with or without presence of DcFLV. The *x*-axis represents the level of statistical differences in DEGs, and the *y*-axis shows the fold change of gene expression (log_2_ fold change). Left red, right red, blue, and black points indicate downregulated (log_2_ fold change <−1), upregulated (log_2_ fold change >1), (−1 ≤ log_2_ fold change ≤ 1), and no significant differences in gene expression, respectively.

**Fig 4:**
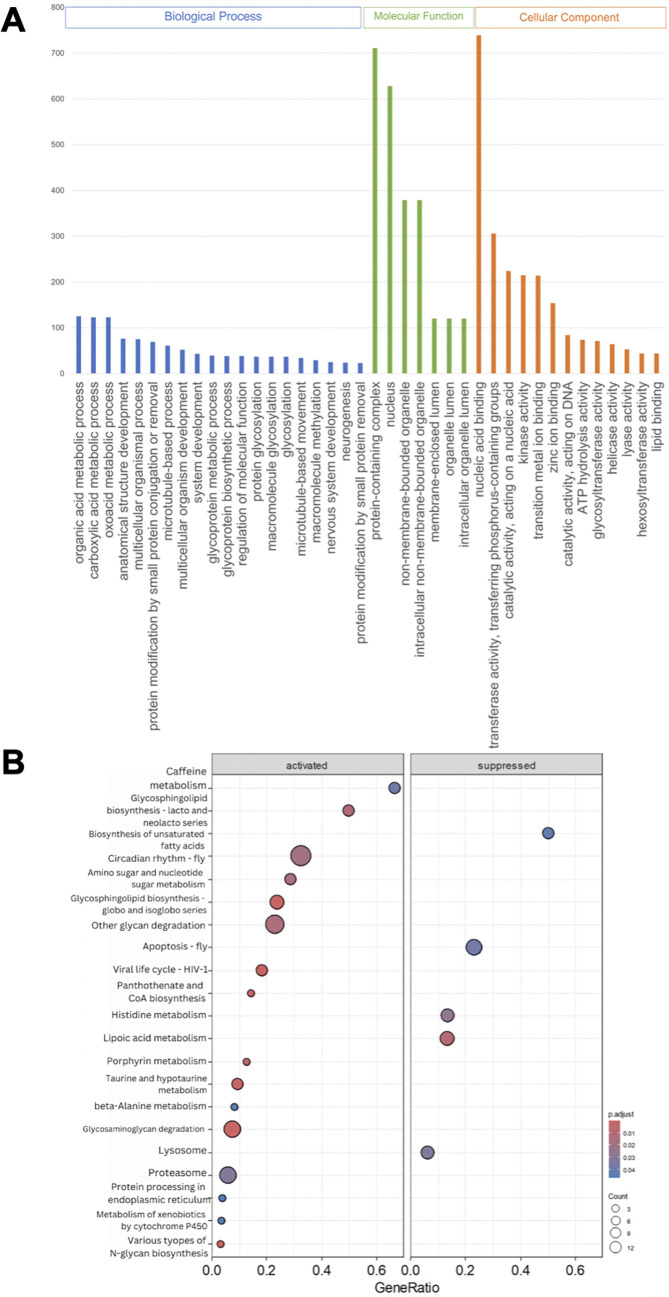
Gene Ontology (GO) classification (**A**) and Kyoto Encyclopedia of Genes and Genomes (KEGG) enrichment analysis (**B**) of DEGs in viruliferous *Diaphorina citri* adults. (**A**) DEGs were grouped into three categories by function: BP (biological process), CC (cellular component), and MF (molecular function). For each category, the top 40 GO terms were selected for functional analysis. The *y*-axis indicates the set sizes of DEGs. (**B**) The top 20 pathways were functionally analyzed by KEGG. The *x*-axis indicates the gene ratio, and the *y*-axis indicates different pathways.

**Fig 5:**
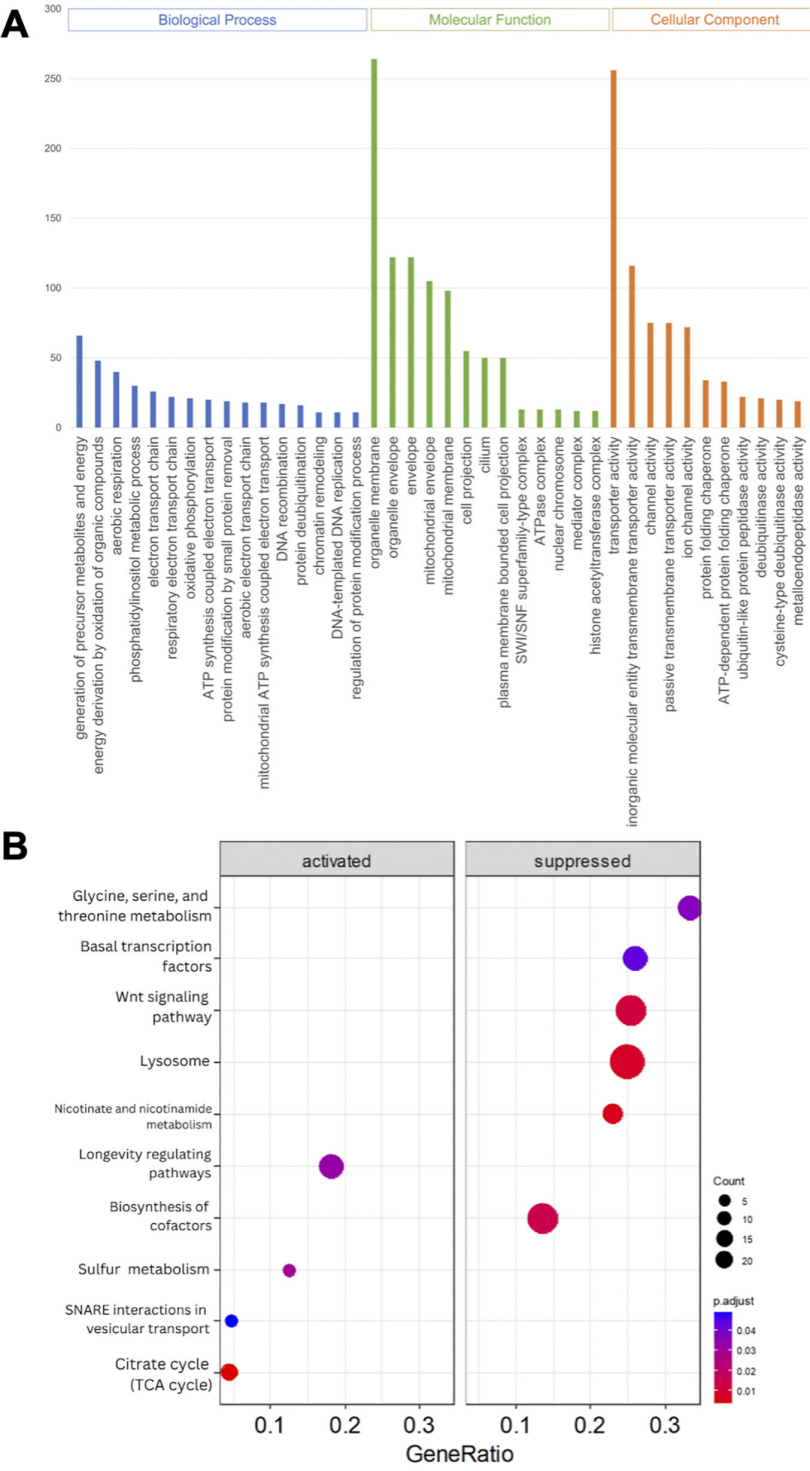
GO classification (**A**) and KEGG enrichment analysis (**B**) of DEGs in viruliferous *Diaphorina citri* nymphs. (**A**) DEGs were grouped into three categories by function: BP (biological process), CC (cellular component), and MF (molecular function). For each category, the top 40 GO terms were selected for functional analysis. The *y*-axis indicates the set sizes of DEGs. (**B**) The top 10 pathways were functionally analyzed by KEGG. The *x*-axis indicates the gene ratio, and the *y*-axis indicates different pathways.

A comparative gene expression analysis between viruliferous and non-viruliferous adults and nymphs was performed using DESeq2, with an adjusted *P* value < 0.05 and |fold change| ≥ 2 as cutoffs. Differential expression analysis indicated that viruliferous adults were characterized by 1,783 upregulated DEGs out of a total of 3,093, whereas infected nymphs exhibited downregulation of 2,197 out of 3,639 DEGs (Fig. 3). Gene ontology (GO) analysis was used to classify DEG functions, with a *P* value < 0.05 indicating significant enrichment. The top 40 significantly enriched GO terms for the three functional categories in infected adults (Fig. 4A) and infected nymphs (Fig. 5A) are provided. To identify active biological pathways, all DEGs were mapped to reference pathways in KEGG. Among all pathways shown in Fig. S1, S2 and S3, viruliferous adults exhibited more active enriched pathways (*n* = 95 out of 149) (Fig. 4B), than viruliferous nymphs (*n* = 44 out of 77 total) (Fig. 5B).

### Greater upregulated expression of endoplasmic reticulum stress, autophagy, and defense-related genes was observed in viruliferous adults than in nymphs

**Fig 6:**
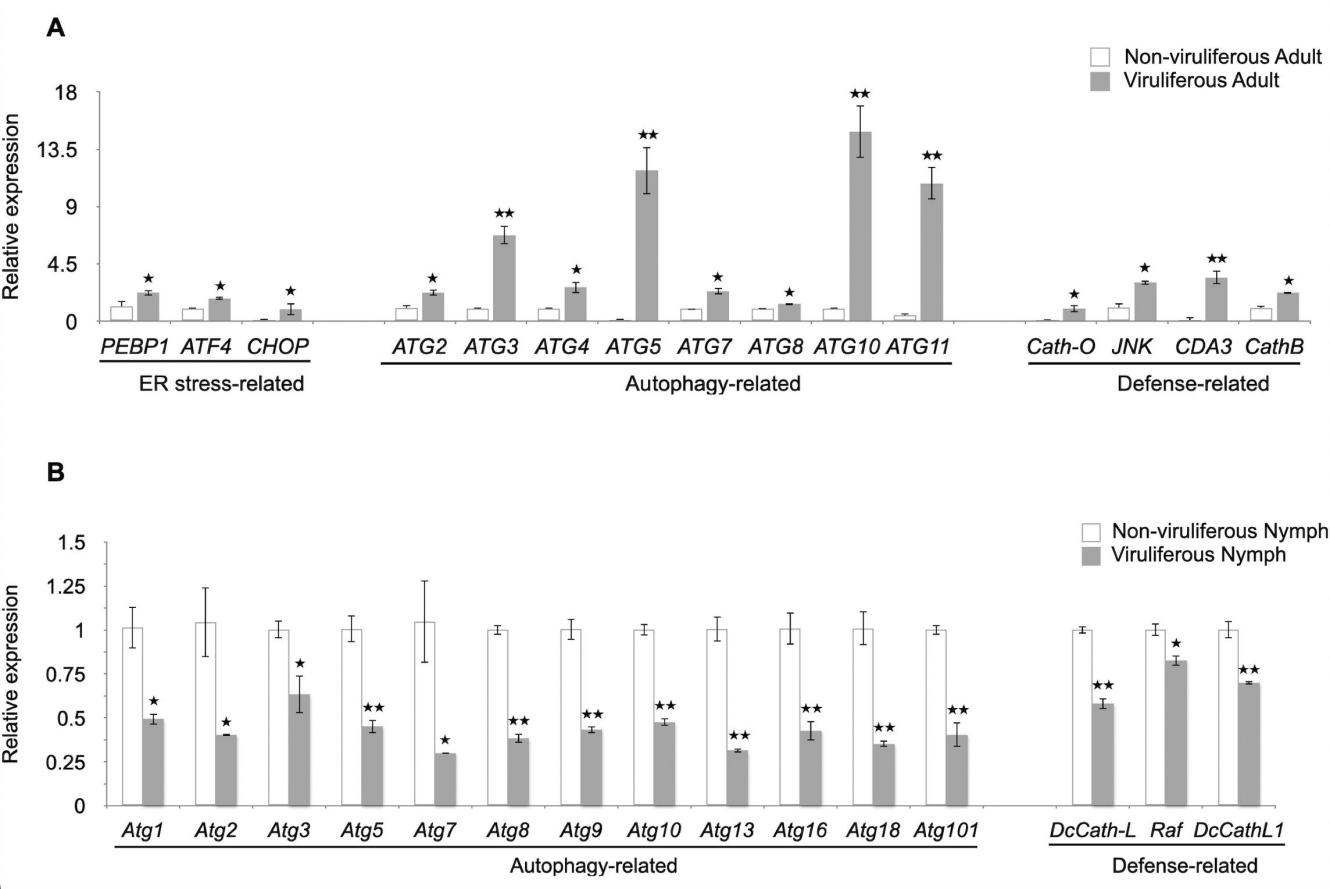
Expression analysis of the functional genes related to ER stress, autophagy, and defense in *Diaphorina citri* adults (**A**) and nymphs (**B**) that were either non-viruliferous or infected with DcFLV as determined by RT-qPCR. Relative expression levels were normalized to the expression levels of the reference gene *DcGAPDH* using the 2^−ΔΔC*t*^ method. Bars represent standard errors, and the asterisks indicate the significant differences (*P < 0.05 or **P < 0.01) as obtained by Student’s *t* tests.

Expression of 5 endoplasmic reticulum stress (ERS)-, 6 apoptosis-, 15 autophagy (ATG)-, and 7 defense (DF)-related genes was compared between viruliferous and non-viruliferous adults and nymphs. All primers that were used for the specific gene identifications are listed in Table S1. In viruliferous adults, 3 out of 5 ERS genes, 8 out of 15 ATG genes, and 4 out of 7 DF genes were upregulated compared with non-viruliferous adult psyllids (Fig. 6A). In contrast, in viruliferous nymphs, 13 out of 15 ATG genes and 3 out of 7 DF genes were downregulated compared to non-viruliferous nymphs (Fig. 6B). Compared to non-viruliferous adults and nymphs, the rest of the function-related genes in viruliferous nymphs and adults showed no statistical differences, except for one apoptosis-related gene (*ALF3*) that was upregulated in viruliferous adults. Moreover, viruliferous nymphs exhibited one downregulated ER stress-related gene (*PEBP1*), two upregulated apoptosis-related genes (*Bcl2* and *TRIAP*), one upregulated autophagy-related gene (*Atg17*), and one upregulated defense-related gene (*DcCath-O*). Overall, most genes involved in ER stress responses, all stages of autophagy, and defense responses were upregulated in viruliferous adults, whereas those genes were downregulated in viruliferous nymphs.

RNA-seq data analysis (Table S2) revealed significant changes in expression levels of 12 differentially expressed genes (DEGs) in viruliferous *D. citri* adults (6 DEGs) and nymphs (6 DEGs) that were not different among non-viruliferous counterparts at the same life stages. The reliability of the RNA-seq data were validated by using qRT-PCR analysis. The gene expression results of these 12 DEGs also confirmed the similar significant changes in expression levels in both viruliferous adults and nymphs (Fig. S4). Presence of DcFLV in adult *D. citri* upregulated an ER stress-related (*ATF4*), antifungal (*MYXVIIIa-like*), serine/threonine-protein phosphatase (*CPPED1*), and chitin synthase (*Chs-2*) gene, whereas it downregulated an ER stress-related (*PERK*) and beta-catenin-like protein 1 gene. In contrast, presence of DcFLV in nymphal *D. citri* upregulated an ER stress- (*eFI2Alpha*), defense-related (*DcCathB*), serine/arginine repetitive matrix protein 1 (*Se/Ar-rm-1*), and trypsin-1-like gene and downregulated an apoptosis-related (*AIF1*) and phospholipid scramblase 1 gene.

## DISCUSSION

HLB is a systemic disease of citrus caused by the bacterial pathogen CLas and there is an urgent need to develop additional strategies for sustainable HLB management beyond vector suppression. Our results indicate that an insect-specific virus (DcFLV) can significantly alter the acquisition and transmission efficiencies of CLas by its *D. citri* vector, thereby potentially impacting epidemiology of pathogen spread. Recently, Roldán et al. (30) described an inverse relationship between abundance of endosymbiotic *Wolbachia* within *D. citri* and its competence as a vector of CLas. Specifically, psyllids in which populations of *Wolbachia* were artificially reduced with antibiotics exhibited higher CLas acquisition as both adults and nymphs as compared with negative controls (30). Increased CLas acquisition was also demonstrated in our current investigation when *D. citri* nymphs became infected with DcFLV. Although the mechanisms mediating the *Wolbachia*-CLas interaction are unknown, potential competitive exclusion between these bacteria was suggested as well as the potential of manipulating the endosymbiont to reduce pathogen spread by the vector (30). By extension, it is possible that manipulating the abundance of DcFLV in field populations of *D. citri* could affect vector competence and associated spread of pathogen. Ammar et al. (12) demonstrated that CLas exploits *D. citri* nymphs mainly for pathogen acquisition and multiplication, and the subsequent adult stage of the vector mainly for pathogen inoculation and spread. Since DcFLV appears to promote the acquisition of CLas at the nymphal stage and transmission of the pathogen by the adult stage of the vector, it appears that pathogen transmission should be overall greater with than without this virus present in populations of *D. citri*. We speculate that reducing the incidence of DcFLV among populations of *D. citri* in commercial citrus groves may benefit HLB management by reducing the transmission of CLas. Although our greenhouse experiment in which infection of citrus plants was more frequent after exposure to vectors co-infected with CLas and DcFLV than to vectors with CLas but without virus supports this hypothesis, field-scale testing will be necessary to verify potential HLB disease management utility.

Insect-associated microbes can cause distinct effects on their hosts depending on the specific insect biotype or genotype that is infected (31, 32). To ensure that biological effects measured here were not attributed to potential variation in insect biotypes, we analyzed genetic similarity among psyllids from our colonies using polymorphic sites in their mitochondrial cytochrome oxidase I (mtCOI) gene. All mtCOI sequences from the psyllid colonies used here (DcFLV/CLas-free and viruliferous only) showed 100% identity to the Dcit-7 haplotype (33), with polymorphism locations consistent across populations when compared to a reference Floridian haplotype (accession number: FJ190176.1) (Fig. S5). These results indicate that all colonies used in this study belonged to the same genotype based on the COI region. Previous studies have also shown that only two haplotypes (Dcit-1 and Dcit-7) of *D. citri* occur in populations found in Florida (33, 34).

**Fig 7:**
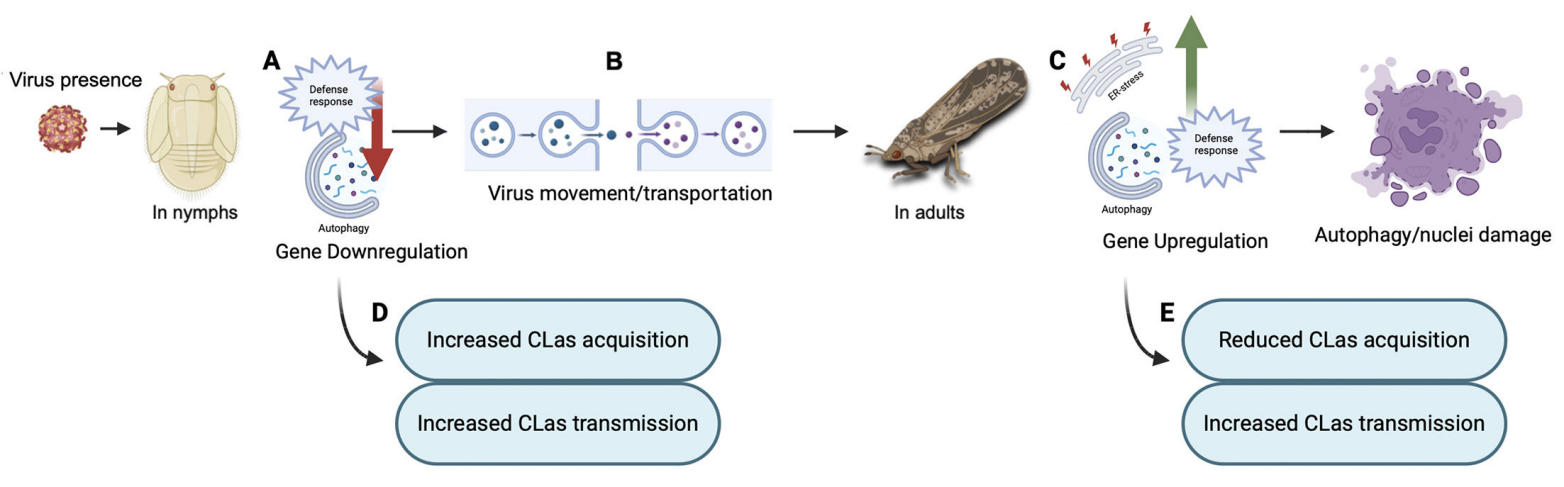
Schematic model of the influence of DcFLV presence on developmental stages of *Diaphorina citri*. During the nymphal stage, (**A**) Viruliferous nymph exhibits downregulated expression of defense- and autophagy-related genes. (**B**) Based on the RNA-seq analysis, viral movement may be encouraged by activating the pathway of vesicular transport activity. At the adult stage, (**C**) the induction of ER stress-, defense-, and autophagy-related genes are observed in viruliferous adults. Microscopic analysis indicates damage to nuclei within gut cells, indicating induction of cell death, which could be related to activation of defense mechanisms. (**D**) During the nymphal stage, DcFLV presence weakens defense responses facilitating both higher acquisition and transmission of CLas. (**E**) During the adult stage, enhanced immune responses inhibit CLas acquisition, but a long-term CLas infection may benefit transmission efficiency by increasing CLas titer. This figure was created with BioRender.com.

Previous studies have addressed the molecular relationships between *Liberibacter* species and their psyllid vectors, suggesting that Liberibacters induce specific functional genes in their psyllid hosts, including those associated with ER stress, autophagy, apoptosis, or defense responses (6, 11, 35–41). These findings elucidated the initial molecular and insect immune responses of psyllids to infection by the Liberibacter phytopathogens they transmit. In the present study, we explored how the presence of DcFLV influences the transmission of a bacterial phytopathogen during both immature and adult stages of development in this hemimetabolous insect vector (Fig. 7) and then applied transcriptomic analysis to gain insight into the possible molecular mechanisms affecting this modulation. Our results indicate that the presence of DcFLV during the nymphal stage suppressed more DEGs (60.4%, 2,197 genes) (Fig. 3) than when virus infection occurred during the adult stage, resulting in fewer enriched pathways in nymphs than adults as determined by GO analysis (Fig. S2). Our analysis of gene expression revealed that genes related to ER stress, autophagy, and defense responses were significantly downregulated in infected nymphs (Fig. 6B), suggesting that these functional genes respond to virus infection (42). It is possible that the presence of DcFLV weakens normal immune responses and reduces autophagic signaling in nymphs, which could benefit the virus by promoting colonization and multiplication at early stages of insect infection (43). Our previous microscopic observations indicated less nuclear damage in gut cells of viruliferous *D. citri* nymphs than adults, which may also explain greater viral colonization of nymphs than adults (15). Our current transcriptomic data suggest that the presence of DcFLV in nymphs may encourage viral movement by activating SNARE-mediated vesicular transport, while simultaneously suppressing defense mechanisms such as lysosomal activity (Fig. 5). During the subsequent adult stage, there was strong activation of defense, ER stress, and autophagic mechanisms (Fig. 6A), linked to induction of cell death reactions including tissue necrosis and nuclear destruction in gut cells (15). The current results, along with those of Kruse et al. (44), suggest that either the psyllid-associated virus or CLas infection can each modulate various aspects of physiological, genetic, and biological pathways in *D. citri* differentially depending on the developmental stage at which the insect becomes infected (45). Future comparative transcriptomic and proteomic data from *D. citri* co-infected with CLas and DcFLV may provide a more comprehensive understanding of the complex molecular interplay among Liberibacter, ISVs, and the psyllid vector during acquisition, latency, and inoculation stages of pathogen transmission.

DcFLV not only localizes with CLas in psyllid gut tissues, but its incidence in *D. citri* populations is also positively correlated with occurrence of CLas (16). Thus, understanding the interaction between DcFLV and the CLas phytopathogen may yield practical insights into whether modification of *D. citri*-associated viruses may affect spread of the bacterial pathogen causing HLB disease. The mechanisms by which DcFLV enhances efficiency of CLas acquisition in nymphs while potentially impeding such acquisition by adults may be related to several factors (Fig. 1). First, our transcriptomic analysis indicates weaker expression of autophagy- and defense-related genes in viruliferous nymphs than in adults as evidenced by a 60% downregulation of DEGs (Fig. 3). This may in part explain why the virus appears to facilitate colonization of *D. citri* nymphs by CLas. In contrast to those effects on nymphs, we observed induction of ER stress, autophagy, and defense responses in viruliferous *D. citri* adults (Fig. 6), which may hinder viral colonization of the adult stage. Furthermore, presence of DcFLV may differentially alter feeding behaviors in *D. citri* nymphs versus adults resulting in differences in acquisition of the CLas bacterium. More detailed observations using electrical penetration graph monitoring will be needed to test this hypothesis. Given that there is less nuclear damage in viruliferous nymphal than in adult gut cells (15), there may be a larger available niche for viral colonization in the immature life stages of the host than in the adult. Finally, differences between nymphal and adult *D. citri* likely exist in terms of the number and/or type of binding sites to CLas (and/or DcFLV) in both the gut and salivary glands (5, 46). These differences may further explain the variations in viral infection observed across different life stages of the host. Future research is needed to test the above hypotheses.

Consistent with previous investigations (10, 47, 48), our results verified that CLas is acquired more efficiently by and multiplies faster in nymphs than adults irrespective of DcFLV presence. Ammar et al. (12) demonstrated that adult *D. citri* more efficiently inoculate citrus hosts with CLas if those adults acquired CLas during early nymphal instars than as adults. These results suggest that high transmission efficiency is dependent on acquisition of CLas during the nymphal rather than adult stage (10). In the current investigation, presence of DcFLV in psyllids significantly increased CLas transmission efficiency by both nymphs and adults compared to non-viruliferous controls (Table 1). The enhanced transmission efficiency could be interpreted in several ways: (i) presence of DcFLV may create beneficial conditions for CLas multiplication in both nymphs and adults, increasing transmission rates because of higher CLas titer (Fig. 2); (ii) viruliferous adults may exhibit higher fitness increasing the latent period of CLas in adults, which may explain higher transmission rates by adults than nymphs. This may also be associated with the requirement that CLas crosses the midgut barrier in order to reach the salivary glands in adults before transmission can occur (49, 50); and (iii) presence of DcFLV may affect salivary gland function in psyllids by modulating pathways related to metabolism, immunity, and cellular processes, which represents an important barrier to CLas transmission (51). In the related psyllid-borne bacterium, *Candidatus* Liberibacter *solanacearum* (CLso), adult potato psyllids (*Bactericera cockerelli*) more efficiently acquire and transmit this pathogen than nymphs (52–54). Evidence from the CLso pathosystem also indicates that transmission efficiency varies depending on psyllid species, CLso haplotype, and vector age (55). In many respects, the CLas and CLso systems have yielded contrasting results illustrating how mechanisms of acquisition and inoculation can differ between different psyllid species serving as vectors of related pathogens. Interestingly, a novel psyllid-associated virus was recently identified from the potato/tomato psyllid (56, 57), and it will be valuable to compare the effects of these different and species-specific, psyllid-associated viruses between *D. citri* and *B. cockerelli*. The current investigation provides a model platform for investigating the interactions among *Liberibacter* species, psyllid-associated viruses, and their psyllid hosts.

In summary, our investigation reveals that the presence of DcFLV manipulates gene regulation in both the nymphal and adult stages of *D. citri*, which facilitates favorable conditions for colonization by and multiplication of the virus in its insect host. Furthermore, presence of DcFLV in *D. citri* differentially modified acquisition and transmission of the CLas phytopathogen; while acquisition of CLas was increased in viruliferous nymphs, transmission was increased in viruliferous adults. Further analyses are needed to determine how infection of *D. citri* with DcFLV may impact vector fitness. Also, a comparative transcriptome analysis of *D. citri* co-infected with CLas and DcFLV deserves further investigation to broaden understanding of the interactions between the bacterial phytopathogen and this insect-associated flavi-like virus. While our laboratory-based experiment indicated that infection of citrus with CLas was greater after exposure to *D. citri* co-infected with CLas and DcFLV than after exposure to vectors with CLas but without virus, field-scale experiments will be necessary to verify whether manipulation of DcFLV could impact HLB disease management.

## MATERIALS AND METHODS

### Maintenance of insects, pathogens, and plants

Three colonies of *D. citri* (DcFLV/CLas-free, viruliferous without CLas, and CLas-infected only without DcFLV) were reared in separate greenhouses at the Citrus Research and Education Center (CREC) in Lake Alfred, Florida. The DcFLV/CLas-free and viruliferous colonies originated from citrus groves in Florida and were maintained on *Murraya koenigii* (curry leaf) plants. The CLas-exposed and presumably CLas-infected *D. citri* colony was originally transferred from DcFLV/CLas-free colony and was reared at CREC on CLas-infected citrus plants (*Citrus macrophylla*) showing HLB symptoms and testing positive for CLas by quantitative PCR (qPCR) (58). Each *D. citri* colony was maintained in insect-proof mesh cages (BugDorm) at 25 ± 2°C under a 14 h light:10 h dark photoperiod and 60–70% relative humidity. To ensure that biological effects measured in this investigation were not caused by potential variation in biotypes of insects used in this study, the polymorphic/biotype of each colony was determined based on the *D. citri* specific mitochondrial cytochrome oxidase I (mtCOI) gene (accession number: FJ190167.1) by using primer sets described previously (DCITRI COI-L/COI-R) (33, 59). Five individual adult psyllids from each colony (DcFLV/CLas-free and viruliferous without CLas) were tested following the protocol in Boykin et al. (33) with slight modification. The amplified products (821 bp) were sequenced, and their genetic relationships were analyzed using the FastME 2.0 method to construct a phylogenetic tree (60). Nymphs from each colony were reared separately from adults in individual mesh cages for subsequent experiments. Representative samples of 20 adults and 20 nymphs from each colony were pooled in groups of five and tested monthly prior to and during experiments to confirm the presence/absence of CLas and DcFLV using qPCR described below.

A CLas-exposed *C. paradisi* (grapefruit) tree was selected from a grove in Florida, and its infected budwood was collected. CLas infection in this tree was confirmed by qPCR as described below. All our CLas-infected plants originated from budwood infection from this individual grapefruit tree. Subsequently, a group of *C. paradisi* plants was graft-inoculated with infected budwood and was maintained in the greenhouse as a CLas resource. The CLas-infected *C. macrophylla* plants used in the study were produced by (i) graft-inoculation with infected budwood from the resource plant and (ii) feeding-inoculation by *D. citri* that acquired CLas from the resource plant. At least 3 months after graft inoculation and confirmation of CLas infection by qPCR, plants were used in experiments or maintained for rearing of CLas-infected psyllids. CLas-free citrus plants were grown from *C. macrophylla* seeds to minimize risk of undetectable infection. Both CLas-infected and CLas-free plants were sampled to confirm CLas infection status by qPCR prior to each experiment. Each group of 6-month-old, CLas-infected and 3-month-old uninfected plants was use in the CLas acquisition and transmission experiments, respectively. Plants were maintained in separate greenhouses to prevent cross-contamination. The rate of CLas infection among *D. citri* adults in the CLas-infected colony ranged between 45% and 80% based on regular qPCR testing of randomly sampled insects during the investigation. In the viruliferous colony, the infection percentage ranged between 60% and 80% based on regular qPCR testing of randomly sampled insects during the investigation as described below. All detection and polymorphic primers used are provided in Table S1.

### Detection of DcFLV and CLas in insect and plant samples

For total DNA extraction, groups of 10 adult or nymphal psyllids were randomly sampled during each experiment from each cage with an insect vacuum (BioQuip Product, Inc., Compton, CA, USA). In addition, 100 mg of midrib tissues were collected from citrus leaves and chopped. Samples were ground in a 2 mL microcentrifuge tube using TissueLyser after dipping in liquid nitrogen. Total nucleic acids were isolated using a modified CTAB method. Insects were immediately placed into liquid nitrogen, and were ground in a homogenizer (Qiagen, Hilden, Germany) for 30 s. Then, 400 µL of CTAB (100 mM Tris-HCl pH 8, 20 mM EDTA pH 8, 1.4 M NaCl, 2% CTAB, 2% PVP, and 0.2% β-Mercaptoethanol) was added and the mixture was incubated at 55°C for 60 min. After incubation, an equal volume of chloroform:isoamyl alcohol (24:1) was added to the homogenate. Samples were vortexed and then centrifuged at 14,000 rpm for 15 min. The aqueous layer was then transferred into a new microcentrifuge tube and 1 vol. of cold isopropanol was added. The tubes were held at −20°C overnight. Total nucleic acids were precipitated at 14,000 rpm for 20 min at 4°C. The pellet was washed with ice-cold 75% ethanol and centrifuged at 14,000 rpm for 5 min. After air drying, total nucleic acids were suspended in 30 µL of double-distilled water, and the quality and yield of DNA was determined using a BioDrop μLite + Microvolume Spectrophotometer (Harvard Bioscience, Inc. Holliston MA, USA). Samples of total nucleic acids were stored at −20°C for further analysis. To detect CLas, qPCR was performed on a 7500 Fast Real-Time PCR system (Applied Biosystems, Waltham, USA) using TaqMan Fast Universal PCR Master Mix (2×) (Applied Biosystem, Waltham, USA) with the primer pair CQULA04F/R and probe CQULAP for the CLas (58). The master mix reaction was performed in 20 µL reaction mixture containing 5 µL of PCR buffer (Applied Biosystem, Waltham, USA), 0.8 µM of each primer (0.2 µL), 0.2 µM of probe (0.1 µL), 100 ng of DNA template (1 µL), and 13.5 µL of water. The qPCR reaction was carried out as follows: 95°C for 2 min; 50°C for 10 min; 40 cycles at 95°C for 15 s, and 64°C for 45 s. Reactions were considered CLas positive when the Ct value was ≤33.

RNA extraction was performed using the sampling method described above. After samples were ground, 400 µL of TRIzol reagent (Thermo Fisher Scientific Inc., Waltham, USA) was added and samples were vortexed. Later, 80 µL of chloroform was added to the homogenate and vortexed for 30 s, and the mixture was incubated at room temperature for 10 min. Samples were centrifuged at 10,000 rpm at 4°C for 15 min. Then, the upper aqueous phase was transferred to a clean 2 mL tube, and 200 µL of cold isopropanol was added and mixed, followed by incubation at RT for 15–60 min. Samples were then centrifuged at 12,000 rpm at 4°C for 15 min. The resulting RNA pellet was washed with 750 µL cold 75% ethanol, and centrifuged at 10,000 rpm at 4°C for 8 min. The air-dried RNA pellet was resuspended in 30 µL RNase-and DNase-free water, and quantity and quality of extracted RNA was measured using nanodrop (Biodrop Ltd., Cambridge, UK), and stored at −80°C. cDNA was synthesized from 0.5 to 1 µg/µL RNA using a high-capacity cDNA reverse transcription kit (Applied Biosystems, Waltham, USA) according to the manufacturer’s instructions, and the resulting cDNA was used as a template. RT-PCR was carried out in 95°C for 2 min, 30 cycles of 95°C for 30 s, 58°C for 30 s, 72°C for 1 min, and a final 72°C extension of 5 min using the primers DcFLVF/DcFLVR (16). PCR products (1,400 bp) were separated in 1% agarose gel staining with GelRed Nucleic Acid Gel Stain (Biotium, Inc., Fremont CA, USA) with 80 vol. for 1 h and visualized in Gel Doc EZ imager (Bio-Rad Laboratories, Inc., Hercules, CA, USA) for the presence of DcFLV. All detection primers used are shown in Table S1.

### CLas acquisition and transmission by viruliferous versus virus-free nymphs and adults

The efficiency of CLas acquisition and transmission was compared between uninfected and viruliferous *D. citri* nymphs and adults. All experiments were performed within separate insect-proof mesh cages (BugDorm) in the greenhouse under the same conditions as described above for insect rearing. For acquisition tests, viruliferous nymphs were obtained from transovarially infected eggs laid by infected females, as DcFLV transmits vertically to offspring (16). Eggs were placed on newly emerged flush shoots of 3-month-old CLas-free *C. macrophylla* plants using forceps and a camel-hair brush under a stereomicroscope and allowed to hatch. The average developmental time of *D. citri* from egg to adult, including five nymphal instars, varies from 14.1 to 49.3 days depending on temperature (61). Fourth-fifth instar nymphs (12- to 14-day old) were transferred and allowed to feed on four replicate 9- to 12-month-old, CLas-exposed *C. macrophylla* plants in groups of five nymphs per plant for 2.5-day (median) AAPs. Thereafter, each group of five psyllid nymphs was collected and preserved at −20°C until the extraction of total nucleic acids for CLas detection with three replicates per group. At least 100–120 viruliferous adults were transferred and fed on CLas-exposed plants for AAPs ranging between 7 and 49 days. AAPs were pooled into seven groups representing 7, 14, 21, 28, 35, 42, and 49 days of feeding. Each group of 10 adults was collected and preserved at −20°C until the extraction of total nucleic acids for CLas detection with three replicates per group. Student’s *t* tests (*P* < 0.05) were used to compare Ct values obtained by qPCR analysis of viruliferous versus non-viruliferous adult or nymphal psyllids after each AAP conducted using the program JMP (v.10, SAS Inc., Cary, NC, USA). The same acquisition protocol was conducted on non-viruliferous *D. citri* adults and nymphs.

For transmission experiments, we used the protocol from Lopes and Cifuentes-Arenas (62) with slight modifications. Fourth-fifth instar nymphs from the non-viruliferous and viruliferous colonies were reared separately on 6-month-old CLas-infected plants in mesh cages for 2.5-day (median) AAPs. Also, adult *D. citri* from the same non-viruliferous and viruliferous colonies were harvested immediately after emergence and allowed 35-day (median) AAPs on 6-month-old CLas-infected plants. Thereafter, each group of nymphs (10 nymphs/seedling; 40–45 seedlings/test) or adults (10 adults/seedling; 25–30 seedlings/test) was transferred to individual 6-month-old, uninfected *C. macrophylla* seedlings for inoculation access periods (IAPs). Each group of CLas-exposed nymphs and adults was allowed 5-day IAPs on uninfected *C. macrophylla* plants. After IAPs, nymphs (*n* = 95) and adults (*n* = 100) were removed and preserved at −20°C until extraction of total nucleic acids for CLas detection. Seedling *C. macrophylla* (labeled by treatment group and dates) were then sprayed with imidacloprid (MINEIRO™2F; Atticus, LLC, Cary NC, USA) and moved to an insect-free greenhouse for subsequent monitoring of CLas infection. Seedlings were observed for symptoms and sampled for infection with CLas by qPCR at 3-, 6-, and 9-month post-inoculation. Student’s *t* tests (*P* < 0.05) were used to compare CLas titers [Log (Copy Number/μL)] between viruliferous and non-viruliferous adult or nymphal psyllids tested by qPCR. Additionally, chi-square tests were conducted to compare the proportion of CLas-infected plants that had been inoculated by CLas-infected *D. citri* with or without the presence of DcFLV. All analyses were performed using the JMP statistical program. The same protocol was conducted on CLas-exposed, but non-viruliferous adults and nymphs.

### RNA-sequencing, assembly, and sequence analyses

We used a slightly modified protocol that was thoroughly described previously (15). Briefly, 250 *D. citri* adults or nymphs were separately sampled at random from the viruliferous and non-viruliferous colonies with an insect vacuum (BioQuip Product, Inc., Compton, CA, USA). Fifty adults and nymphs from each psyllid population were then sub-sampled and collected into individual extraction tubes and total RNA was extracted for RNA-seq analysis using TRIzol reagent (Thermo Fisher Scientific Inc., Waltham, MA, USA) according to the manufacturer’s instructions. Prior to sequencing, each group of viruliferous or non-viruliferous psyllid adults or nymphs was analyzed by qPCR, as described above, to confirm infection status with DcFLV. In addition, CLas infection status was also confirmed by qPCR for those groups of psyllids presumed to be either CLas-free or infected prior to subsequent RNA-sequencing. The extracted RNA samples from adult (avg. 940 ng/µL) and nymphal psyllids (avg. 1,300 ng/µL) were verified for sufficient purity and quality by determining the RNA integrity number (RIN) using an Agilent 2100 Bioanalyzer (Agilent Technologies, Palo Alto, CA, USA) and a BioDrop Spectrophotometer (Biochrom Ltd., Cambridge, UK). RNA samples with a RIN of 8.0 or greater, a 260/280 ratio of 1.9 or greater, and a 260/230 ratio of 2.0 or greater were used for further analysis. Four RNA sample types were obtained (viruliferous nymphs, viruliferous adults, non-viruliferous nymphs, and non-viruliferous adults) with three biological replicates for each group (a total of 12 RNA samples). Samples were sent to the University of Florida’s Interdisciplinary Center for Biotechnology Research (UF-ICBR). Aliquots of the total RNA samples were separated, and ribosomal RNA depletion as well as the construction of a cDNA library were performed using the SMART kit from Clontech (Mountain Views, CA, USA). The generated cDNA library was normalized using the Trimmer kit from Evrogen (Evrogen Joint Stock Company, Moscow, Russia) following the manufacturer’s instructions. All four groups of samples were sequenced on the Illumina NovaSeq6000 (Illumina, San Diego, CA, USA) by UF-ICBR. Sequencing was performed as described by Margulies et al. (63) with slight modifications. Three biological replicates of each treatment for each insect stage were used. Certain steps of the data analysis were performed as described in Mann et al. (64). First, the raw mRNA reads were checked for anomalies and adapters using FastQC, followed by the removal of Illumina universal adapters using AdapterRemoval. The output file was analyzed with FastQC and SortMeRNA, which removed rRNA based on rRNA databases and provided two output types: reads that mapped to rRNA and those where one or both PE reads did not map to rRNA. Trimmomatic was then used to remove sequences and paired reads where one or more were shorter than 17 nucleotides. The *D. citri* (GCF_000475195.1_Diaci_psyllid_genome_assembly_version_1.1_genomic.fna) genome was indexed using HISAT2, and the cleaned reads were aligned to the indexed genomes using HISAT2. The concordantly aligned reads were checked with FastQC, converted to BAM, and sorted by name using SAMtools. StringTie was used to bundle the reads into transcripts and align them to the GTF/.GFF file for the *D. citri* genome. A count matrix was formed using StringTie, Ballgown, and the prepDE.py3 script to allow downstream differential expression (DE) analysis using the default procedure with DESeq2 in R. DEGs were identified using a threshold of an adjusted *P* value of 0.05 and a minimum fold change of 2 and −2. Finally, gene set, and pathway enrichment analysis was developed using the AnnotationHub, clusterProfiler, DOSE, enrichplot, ggridges and pathview libraries from the Bioconductor project in R. The R code and the scripts used for the bioinformatic analysis are available in this publicly available github repository: https://github.com/jrobledob/Transcriptomics_DCFLV_Lin_et_al_2024 (repo now available).

### Absolute quantification of CLas

The preparation of standard DNA templates for standard curve construction is described below. The whole sequence for “ribosomal protein L10 (rplJ)” (703 bp) was cloned into pGEMT-easy vector system (Promega Corporation, Madison, WI, USA). Standard dilutions were prepared for known concentration of purified plasmid starting with 4.908E + 10 copies/µL and serial dilutions were made to produce an eightfold, 10-point dilution series. The primer sequences and probe (Table S1) targeting ribosomal protein L10 (rplJ) (87 bp) of CLas were used to detect amplification (58). Each reaction was run in duplicate with the thermo cycling profile was comprised of an initial 10 min denaturation step at 95°C and 40 cycles of 95°C for 15 s and 64°C for 45 s with fluorescent data collection during the 64°C step. Data acquisition was accomplished by Applied Biosystems, Waltham, USA. The standard curve was designed by plotting Ct values against Log10 values of plasmid copy numbers used as the template. Absolute copy number in each sample was estimated by using regression equation from the standard curve: *y* = 4.1764 *x* + 4.0344, *R*^2^ = 0.9986 (Copy = antilog10 (Ct − *a*)/*b*; where *a* = 48.68; *b* = −4.18) (Fig. S6). For each qPCR test, a plasmid containing the partial fragment of the CQULA gene (703 bp) was used as a positive control. The Ct value of the control plasmid was between 22 and 26, which was adjusted to 24 ± 0.5000 and used as the calibration to all unknown samples for both CLas acquisition and transmission experiments.

### Relative quantification of specific genes

Expression of ERS-, apoptosis-, autophagy-, and defense-related genes in viruliferous or non-viruliferous nymphs and adults was quantified by quantitative reverse transcription polymerase chain reaction (RT-qPCR) (three biological replicates for each treatment). PowerUp SYBR Green Master Mix (2×) (Applied Biosystem, Waltham, USA) was used to carry out RT-qPCR following the manufacturer’s instructions. The RNA extraction and cDNA synthesis protocols were done as described above. The concentration of each RNA and cDNA sample was determined with a BioDrop spectrophotometer, and then each cDNA sample was diluted to 100 ng/µL for the qPCR. The reaction mixture consisted of 100 ng of cDNA and 300 nM of each gene-specific primer in a final volume of 10 µL. The *D. citri GAPDH* gene was used as the reference gene. The 2^−ΔΔC*t*^ method (65) was used to calculate the expression levels of these genes. Student’s *t* tests were used to compare gene expression at a *P <* 0.05 confidence level. To further validate the reliability of the RNA-seq data comparing viruliferous and non-viruliferous adults and nymphs, three specific functional genes related to ER stress, apoptosis, and defense, as well as three randomly selected genes, were chosen from both adults and nymphs. Statistical analysis of gene expression obtained with qRT-PCR was conducted as described above and used to compare the expression results with the RNA-seq data for these 12 specific genes (Table S2). All gene-specific primers used in this study are provided in Table S1.

## Data Availability

The RNA-Seq data are available in the NCBI sequence read archive (SRA) with accession number PRJNA1103173. SRR28769332 and SRR28769333 correspond to viruliferous and non-viruliferous treatments for nymphs. SRR28769334 and SRR28769335 correspond to viruliferous and non-viruliferous treatments for adults. All data supporting the findings of this study are available within the paper and are available from the corresponding author upon request.

## SUPPLEMENTAL MATERIAL

Supplemental material jvi.01630-24-s0001.docx Table S1; Figures S1 to S6.

Table S2 jvi.01630-24-s0002.docx Summary of all differentially expressed genes in viruliferous Diaphorina citri adults and nymphs.

Table S3 jvi.01630-24-s0003.xlsx Summary of all Gene Ontology (GO) annotation analysis in viruliferous Diaphorina citri adults and nymphs.

Table S4 jvi.01630-24-s0004.xlsx Summary of all Kyoto Encyclopedia of Genes and Genomes (KEGG) enrichment analysis in viruliferous Diaphorina citri adults and nymphs.

ASM does not own the copyrights to Supplemental Material that may be linked to, or accessed through, an article. The authors have granted ASM a non-exclusive, world-wide license to publish the Supplemental Material files. Please contact the corresponding author directly for reuse.
