# Supplementary material for: An insect virus differentially alters gene expression among life stages of an insect vector and enhances bacterial phytopathogen transmission
Source: J Virol. 2024 Dec 23;99(1):e01630-24. doi: 10.1128/jvi.01630-24 (PMC11784072; doi:10.1128/jvi.01630-24)

**TABLE S1** Primers and probes used for pathogens detection, analysis of *Diaphorina citri* population diversity, and absolute/relative quantification of selected genes by qPCR

| Target^a^ | Sequence (5’-3’) | Product Size (bp) | Reference |
| --- | --- | --- | --- |
| DcFLVF | AGGCGAGTACTCCCATCGGATACATT | 1400 | (16) |
| DcFLVR | GAGGGCCGCTAAGTCTGTAGGACATATT |  |  |
| DCITRI COI-L | AGGAGGTGGAGACCCAATCT  TCAATTGGGGGAGAGTTTTG | 821 | (33, 59) |
| DCITRI COI-R |  |  |  |
| CLas (CQULA04F) | TGGAGGTGTAAAAGTTGCCAAA | 87 | (58) |
| CLas (CQULA04R) | CCAACGAAAAGATCAGATATTCCTCTA |  |  |
| CQULAP | 6FAM-ATCGTCTCGTCAAGATTGCTATCCGTGATACTAG |  |  |
| PEBP1-F | CAACCACTCCACATGGCTGA | 64 | This study |
| PEBP1-R | CGAGTTGGCAAGACAGAGCC |  |  |
| PERK-F | GGCCAACTCAGCCTCGTCA | 141 |  |
| PERK-R | TCTTTGGGTTAGTACGGGCG |  |  |
| eIF2α-F | AGCTGAGATGGGTGCCTATG | 195 |  |
| eIF2α-R | GCCTTCTCTGGAGTGACAGT |  |  |
| ATF4-F | GCGGCGCTACCTATTGAAGA | 170 |  |
| ATF4-R | CGGCTCAGATCTTCCACACT |  |  |
| CHOP-F | GCGCGAGCGAAATAACATAGC | 186 |  |
| CHOP-R | TCAGGCAGTACTCCTACATTGGT |  |  |
| Bcl2-F | AGGAGGCTTTGTGATTCGGG | 229 |  |
| Bcl2-R | AGCCACACAGTACAGGGAGA |  |  |
| IAP-F | CCGCCAGCATTCCCTAACTA | 162 |  |
| IAP-R | CAAACCTCCACCACATCGGA |  |  |
| AIF1-F | GGTGTGGGACAAGCACTACT | 218 | This study |
| AIF1-R | CCGCTGTAGTTTTGGTGGAC |  |  |
| AIF3-F | TGCTGTTGGAACCAAATGCTC | 151 |  |
| AIF3-R | ACAAGGGAGACTGTCCATGC |  |  |
| TRIAP-F | ACCTCCAAGCTAAAAAGAGCAA | 202 |  |
| TRIAP-R | CCCATCATGAGTTGATGGAAAGT |  |  |
| IAPP2-F | ATCACCGAGTACCCAAGCAC | 190 |  |
| IAPP2-R | TCCATAGCTGTCCACTTGCG |  |  |
| IAPP5-F | AGTGGTAACTGTACTGCAGAG | 164 |  |
| IAPP5-R | TGGACAAAAGGACAGTCTGG |  |  |
| IAPP6-F | CCCACCTACCTCCAGCCT | 211 |  |
| IAPP6-R | GGCCGGATGTAGCACATAGA |  |  |
| Atg1-F | TTGGCAAACAGTACCCCCAG | 105 | (6) |
| Atg1-R | AACCACAAAGTGTTGCTGCC |  |  |
| Atg2-F | ATACGAACCCTTGCCCATCG | 104 |  |
| Atg2-R | CCAGCGATATCAGAGCGGAG |  |  |
| Atg3-F | AGGTCTGTGCCATTCAGAGC | 85 |  |
| Atg3-R | AAACGTGATATTGATGCTCACC |  |  |
| Atg4-F | TGAACAGGGACCAAGACTTCC | 80 |  |
| Atg4-R | TTGCCAATCCCGACCTGAAA |  |  |
| Atg5-F | TGATCTGTGGTGGACTGTGC | 98 |  |
| Atg5-R | GGGAACGGGCCTTTACAAGA |  |  |
| Atg6-F | CTTTGGTGCTTCGCCTGTTC | 115 |  |
| Atg6-R | ATTTGGGGATCTTGCTGGGG |  | (6) |
| Atg7-F | ACAAGCAATGGAGGATGGTGA | 118 |  |
| Atg7-R | TGCAGGAAACCCAAGCCAATA |  |  |
| Atg8-F | TGCGTACAGTGACGAGAACG | 74 |  |
| Atg8-R | TCCAAGGGTTTGCAGACTGG |  |  |
| Atg9-F | GTGGGTGAGCTATTCCGGTT | 110 |  |
| Atg9-R | GATGTGATGCCAGGGGGTAG |  |  |
| Atg10-F | GTTGCATCGCGTGCTATGTT | 74 |  |
| Atg10-R | TTCAGCACCCAATGCTGTCA |  |  |
| Atg13-F | CTGGTACCAAACTGACCCCC | 117 |  |
| Atg13-R | TCGTGTAGACTTTGGCACCC |  |  |
| Atg16-F | TGTGTGCCAAATTCCTGGGT | 111 |  |
| Atg16-R | CCGCGAACTTCGTCTCTGTA |  |  |
| Atg17-F | CGTGCTCGCATTTTGTCGAT | 119 |  |
| Atg17-R | GAAATTCCCGAGTGCGGAGT |  |  |
| Atg18-F | AGCAACACGCCAATAGTCCA | 76 |  |
| Atg18-R | CCTCCGTTAATCTTGGGCGT |  |  |
| Atg101-F | GACCAACAAAGTGAGCGCAA | 73 |  |
| Atg101-R | ATCAACAAGCGCTGTGAAGC |  |  |
| DcCath-L-F | TCTTTCCACCGCCGAGTTC | 208 | (66) |
| DcCath-L-R | CACGCCTTCAATGTTTCCTGTC |  |  |
| DcCath-O-F | TGTGGCGGTGATAGCGTTGT | 205 |  |
| DcCath-O-R | CTCGGGAGATTGGCGGTTT |  |  |
| JNK-F | GACGCCCTCATGCATCCTTA | 194 | This study |
| JNK-R | CCGCGTCTTTCCATTGGTCT |  |  |
| Raf-F | GAGCAAGTGGTCAGGAGGTC | 87 |  |
| Raf-R | GGCTCTTGCATGCGAATCAC |  |  |
| DcCDA3-F | CAGATGAGGTCTTGGAGTGGCT | 222 | (67) |
| DcCDA3-R | GTTCTTTGCTTGACTTTGGGTTG |  |  |
| DcCathB-F | CTGGACGAATCAGACGAGGA | 136 | This study |
| DcCathB-R | AGCCCATGGTTTCCAGATCA |  |  |
| DcCathL1-Fw | CCACCATTGGACCCGTATCT | 85 |  |
| DcCathL1-Rv | TCAGGCTCGTAGTACACACC |  |  |
| myXVIIIa-like-F | AAGAGCTCACATTCTCCGGC | 133 |  |
| myXVIIIa-like-R | CGCTCCCTGACTTACCTGTC |  |  |
| CPPED1-like-F | TGGCACGGGGATTTCAGTTT | 73 |  |
| CPPED1-like-R | TCGGATGTTCAAACTGAGCGA |  |  |
| Chs-2-like-F | TGAAGAAGGCAGTCTGGAGC | 81 |  |
| Chs-2-like-R | TTCTCATCCACTGGCTTGGG |  |  |
| cDKI 1C-like-F | TCCAAGATGTTCCCACCACC | 84 |  |
| cDKI 1C-like-R | CAACGAAAGTTGGTTTGGCA |  |  |
| b-catenin-like-F | TGAAGCGGATAAGGAGCAGC | 135 |  |
| b-catenin-like-R | TTAATAGTGGCAGCCCCAGC |  |  |
| Se/Ar-rm-1-F | AGGTCGAGATACGAACGGAGA | 77 |  |
| Se/Ar-rm-1-R | CACCATCACGAGAGGAAGCA |  |  |
| Trypsin-1-like-F | TAGAGCAGGTAGGCCTTGGT | 71 |  |
| Trypsin-1-like-R | GGACGCCACCACAGAAGTTA |  |  |
| Ps1-F | TCGATATTCCATGTGCGGGG | 76 |  |
| Ps1-R | AATACGCCCCACTTCGGTTT |  |  |
| Hedgehog-like-F | CAAGAACGGCAAGGCGTATG | 82 |  |
| Hedgehog-like-R | TGTCGTCAGAGGAGCGTAGA |  |  |
| DcGAPDH-F | GACACTCACTCCTCCATCTTT | 88 | (6) |
| DcGAPDH-R | GTATCCGTACTCGTTGTCATACC |  |  |

^a^ PEBP1: *D. citri* phosphatidylethanolamine-binding protein 1 (XM_008488434.3); PERK: *D. citri* eukaryotic translation initiation factor 2-alpha kinase 3-like (XM_026822487.1); eIF2Alpha: *D. citri* eukaryotic translation initiation factor 2 subunit 1 (XM_026826819.1); ATF4: *D. citri* cyclic AMP-dependent transcription factor ATF-4 (XM_017449302.2); CHOP: *D. citri* CCAAT/enhancer-binding protein-like (XM_026823321.1); Bcl2: *D. citri* LOC103517186 (XM_026829051.1); IAP: *D. citri* putative inhibitor of apoptosis (XM_008471116.3); AIF1: *D. citri* apoptosis-inducing factor 1 (XM_017448352.2); AIF3: *D. citri* apoptosis-inducing factor 3 (XM_008488216.1); TRIAP: *D. citri* TP53 regulated inhibitor of apoptosis 1 (XM_008469737.3); IAPP2: *D. citri* death-associated inhibitor of apoptosis 2 (XM_026823088.1); IAPP5: *D. citri* baculoviral IAP repeat-containing protein 5 (XM_017447232.2); IAPP6: *D. citri* baculoviral IAP repeat-containing protein 6 (XM_026825043.1); JNK: *D. citri* stress-activated protein kinase JNK (XM_026823423.1); Raf: *D. citri* serine/threonine-protein kinase A-Raf-like (XM_008488867.2); DcCathL1: *D. citri* cathepsin L-like protein (MN_166228.1); myXVIIIa-like: *D. citri* unconventional myosin-XVIIIa-like (XM_008488296.2); CPPED1-like: *D. citri* serine/threonine-protein phosphatase CPPED1-like (XM_008479591.3); Chs-2-like: *D. citri* chitin synthase chs-2-like (XM_017447570.2); cDKI 1C-like: *D. citri* cyclin-dependent kinase inhibitor 1C-like (XM_008482306.1); b-catenin-like: *D. citri* beta-catenin-like protein 1 (XM_017447891.2); Se/Ar-rm-1: *D. citri* serine/arginine repetitive matrix protein 1 (XM_026823678.1); Trypsin-1: *D. citri* trypsin-1-like (XM_026831816.1); Ps1: *D. citri* phospholipid scramblase 1 (XM_008471781.3); Hedgehog-like: *D. citri* protein hedgehog-like (XM_026826587.1)

References:

66. Yu H-Z, Huang Y-L, Li N-Y, Xie Y-X, Zhou C-H, Lu Z-J. 2019. Potential roles of two Cathepsin genes, DcCath-L and DcCath-O in the innate immune response of *Diaphorina citri*. J Asia Pac Entomol 22:1060–1069. <https://doi.org/10.1016/j.aspen.2019.05.010>.

67. Yu HZ, Li NY, Li B, Toufeeq S, Xie YX, Huang YL, Du YM, Zeng XD, Zhu B, Lu ZJ. 2019. Immune functional analysis of chitin deacetylase 3 from the Asian citrus psyllid *Diaphorina citri*. IJMS 21:64. <https://doi.org/10.3390/ijms21010064>.

**TABLE S2** Summary of all differentially expressed genes in viruliferous *Diaphorina citri* adults and nymphs


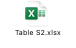


**TABLE S3** Summary of all Gene Ontology (GO) annotation analysis in viruliferous *Diaphorina citri* adults and nymphs


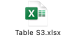


**TABLE S4** Summary of all Kyoto Encyclopedia of Genes and Genomes (KEGG) enrichment analysis in viruliferous *Diaphorina citri* adults and nymphs


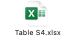


**FIG S1** Summary of all Gene Ontology (GO) annotation of DEGs in viruliferous *Diaphorina citri* adults. DEGs were grouped into three categories by function: BP (biological process), CC (cellular component), and MF (molecular function). The *y*-axis indicates the set sizes of DEGs


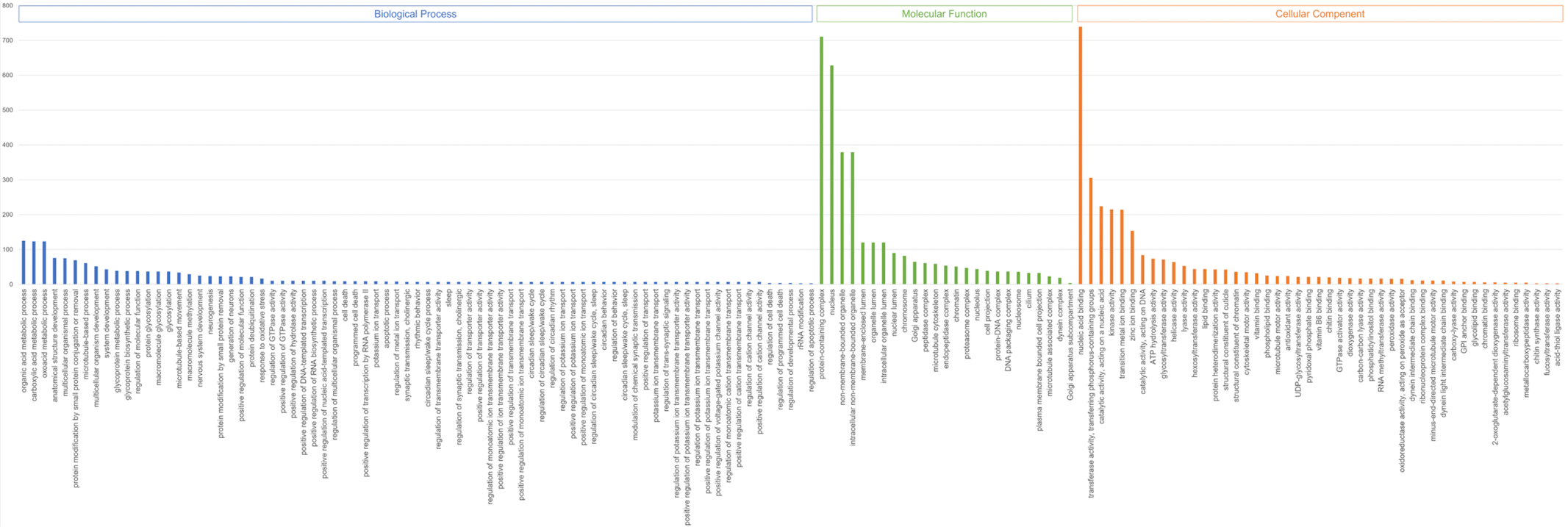


**FIG S2** Summary of all Gene Ontology (GO) annotation of DEGs in viruliferous *Diaphorina citri* nymphs. DEGs were grouped into three categories by function: BP (biological process), CC (cellular component), and MF (molecular function). The *y*-axis indicates the set sizes of DEGs


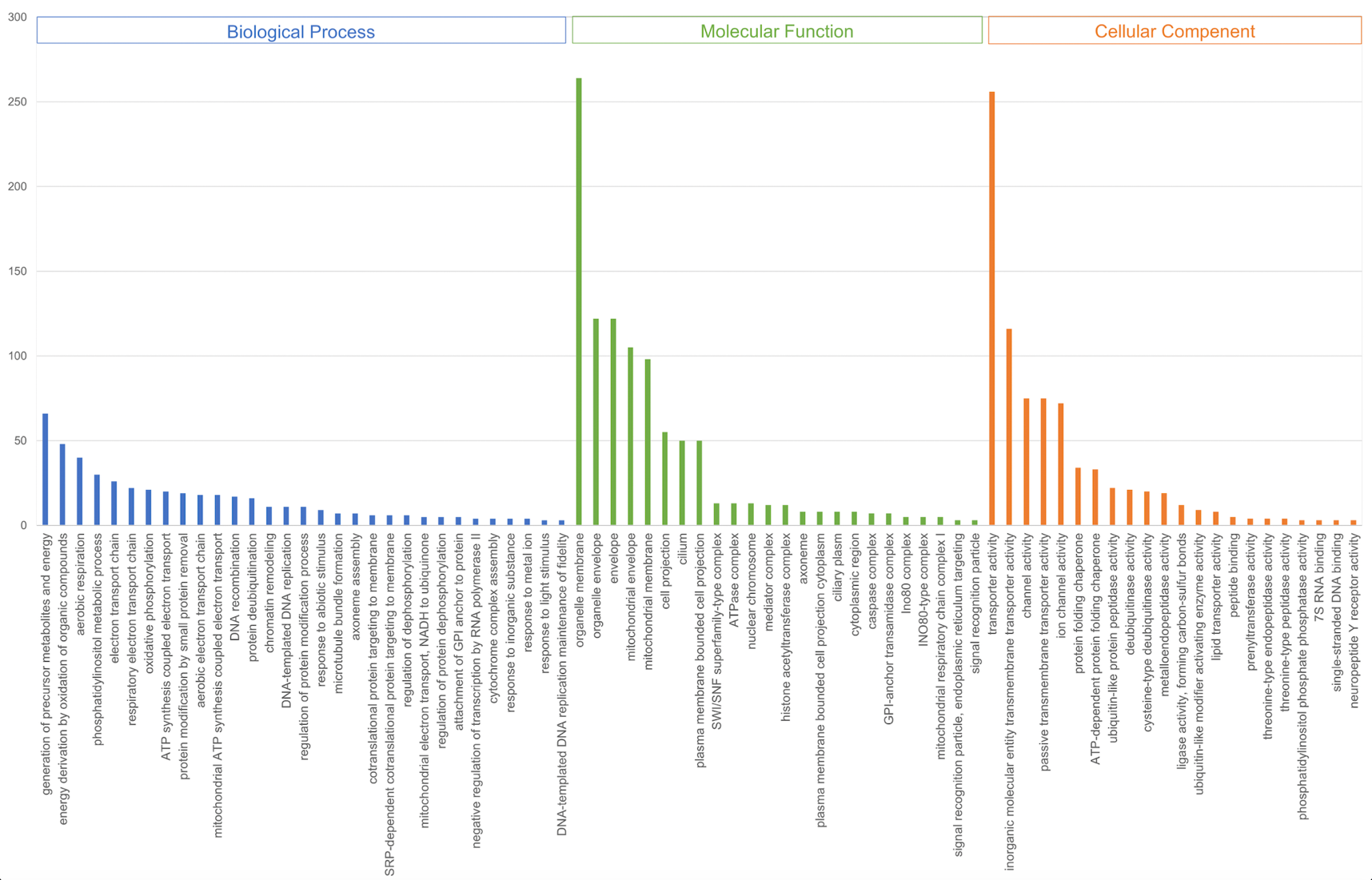


**FIG S3** Kyoto Encyclopedia of Genes and Genomes (KEGG) enrichment analysis of DEGs in *Diaphorina citri* adults (A) and nymphs (B) that were infected with DcFLV

**FIG S4** Expression pattern of specific functional genes related to ER stress, apoptosis, and defense in DcFLV viruliferous *Diaphorina citri* adults (**A-F**) and nymphs (**G-L**) compared to non-viruliferous adults and nymphs using RNA-seq transcriptome data and reverse transcription quantitative PCR (RT-qPCR). For RT-qPCR, twelve genes related to specific functions were selected, and expression levels of tested genes were normalized based on *D. citri GAPDH* gene. Asterisks represent significant differences by evaluating with Student’s *t*-test (**P <* 0.05), and error bars represent standard deviations of the means


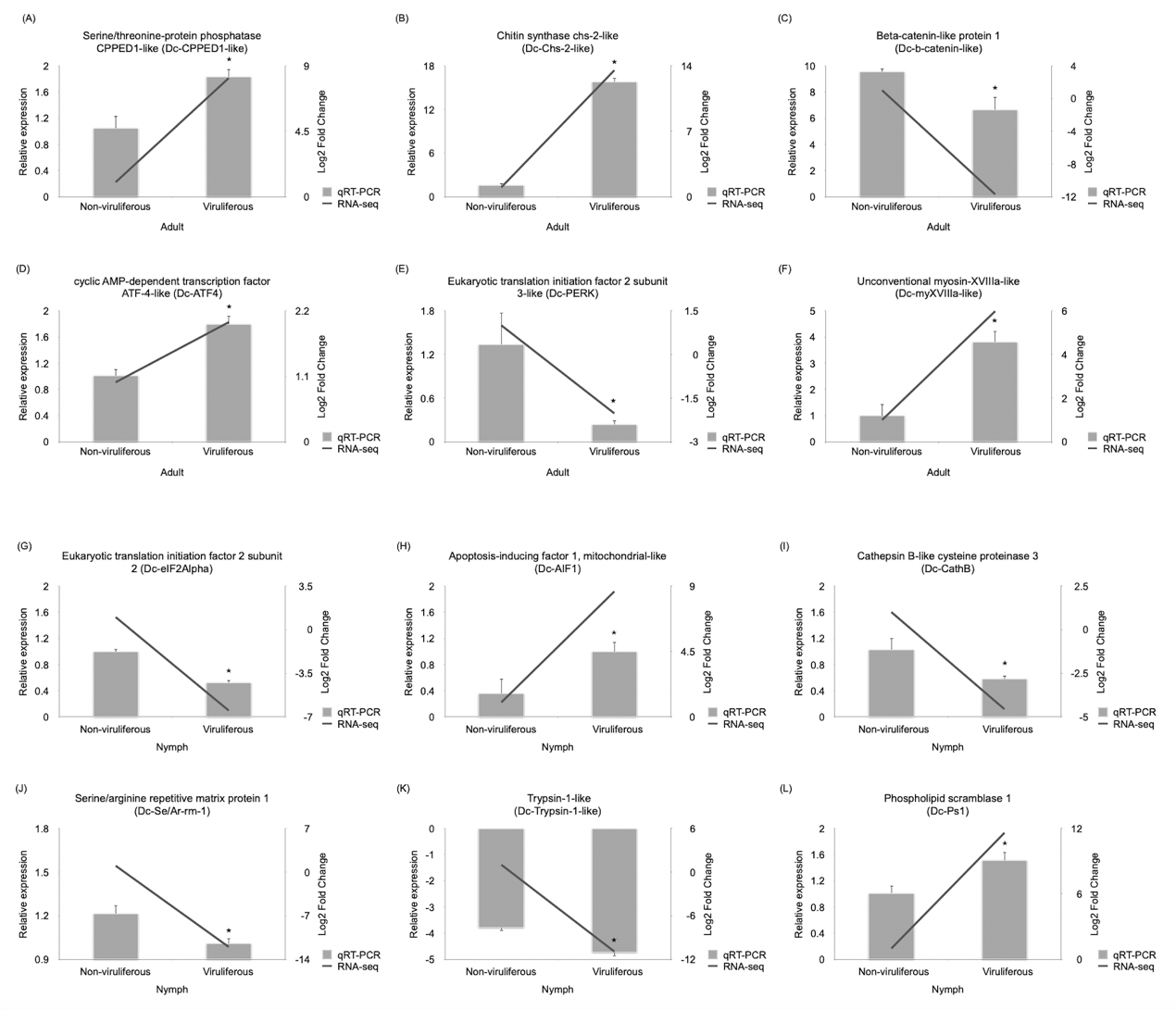


**FIG S5** Phylogenetic tree of the mitochondrial cytochrome oxidase I (mtCOI) gene of *Diaphorina citri*. The sequences were obtained from regular PCR and were aligned using MUSCLE. The phylogenetic tree was inferred by using FastME 2.0. Mitochondrion gene of *Pachypsylla venusta* (Accession number: NC_006157.1) was used as an outgroup to root the trees. The number at each branch of the phylogenetic tree represents the bootstrap value


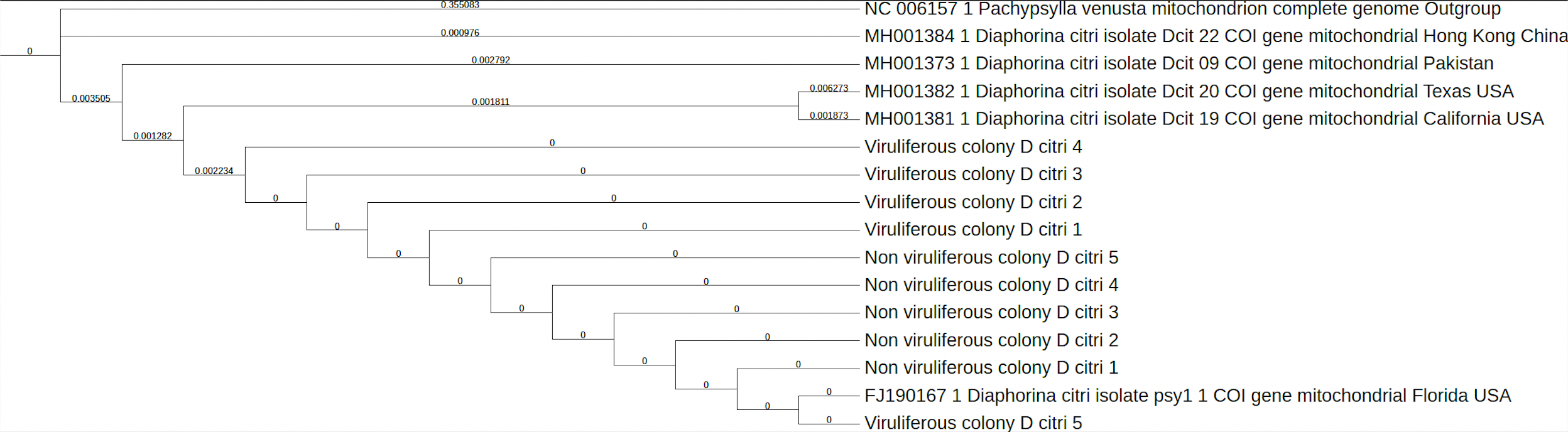


**FIG S6** Standard curve of qPCR assay with tenfold serially diluted pGEM®T-easy vector contained partial sequence of CLas ribosomal protein L10 using CQULA primers


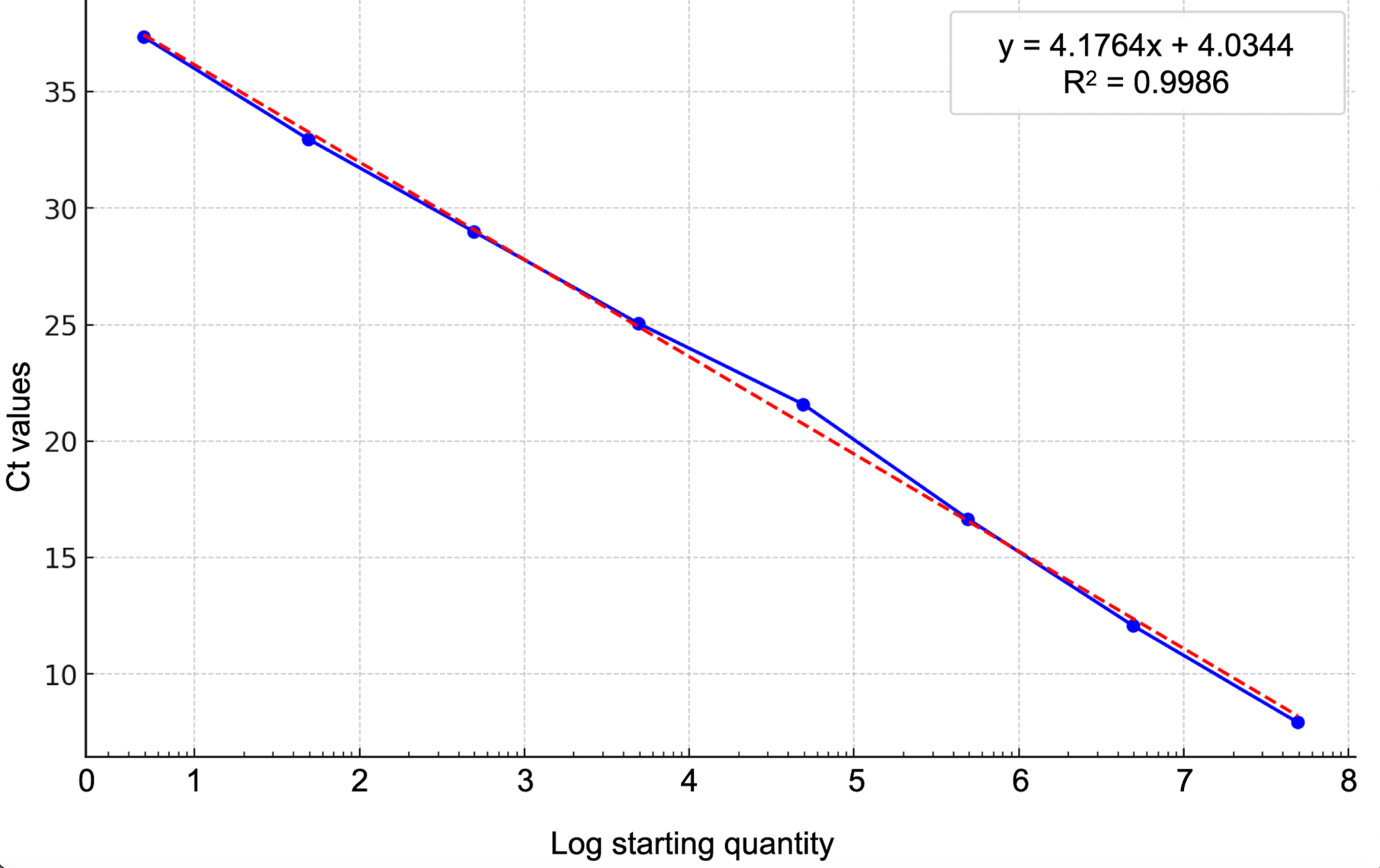

Supplement: Supplemental material — Table S1; Figures S1 to S6. [file jvi.01630-24-s0001.docx]
